# Body mass index is associated with higher Gleason score and biochemical recurrence risk following radical prostatectomy in Chinese men: a retrospective cohort study and meta-analysis

**DOI:** 10.1186/s12957-015-0725-0

**Published:** 2015-11-05

**Authors:** Pei-De Bai, Meng-Bo Hu, Hua Xu, Wen-Hui Zhu, Ji-Meng Hu, Tian Yang, Hao-Wen Jiang, Qiang Ding

**Affiliations:** Department of Urology, Huashan Hospital, Fudan University, No.12 Middle Wulumuqi Road, Shanghai, 200040 China

**Keywords:** Prostatic neoplasms, Body mass index, Prostatectomy, Biochemical recurrence, Gleason score

## Abstract

**Background:**

The aim of the study is to investigate whether body mass index (BMI) affected pathological characteristics and biochemical recurrence (BCR) of prostate cancer after radical prostatectomy in Chinese men.

**Methods:**

Medical records of 211 Chinese patients who underwent radical prostatectomy between 2006 and 2014 were retrospectively reviewed, with follow-up time of 24.5 ± 27.0 months. Multivariate logistic and Cox regression analyses were applied to address the impact of BMI on adverse pathological outcomes and BCR following prostatectomy. A meta-analysis of published studies from MEDLINE or EMBASE was conducted to determine the relationship between BMI and BCR following prostatectomy among Asian populations.

**Results:**

Higher BMI was positively correlated with higher biopsy Gleason score (odds ratios (OR) 1.163, 95 % confidence interval (CI) 1.023–1.322, *P* = 0.021) and pathological Gleason score (OR 1.220, 95 % CI 1.056–1.410, *P* = 0.007) in multivariate analysis. BCR was detected in 48 patients (22.7 %). Multivariate Cox proportional hazards analysis revealed that higher BMI (hazard ratio (HR) 1.145, 95 % CI 1.029–1.273, *P* = 0.013) and prostate-specific antigen (HR 1.659, 95 % CI 1.102–2.497, *P* = 0.015) levels were independent predictors of BCR. The meta-analysis enrolled eight Asian studies of 4145 patients treated by radical prostatectomy. Based on random-effects approach, a 5 kg/m^2^ increase in BMI was correlated with 28 % higher risk of BCR (HR 1.22, 95 % CI 0.86–1.72) without statistical significance.

**Conclusions:**

The present study suggested that higher BMI was an independent risk factor for a higher Gleason score, as well as an independent predictor of BCR after radical prostatectomy in Chinese patients. Meta-analysis of Asian studies also indicated that obese patients, although without statistical significance, might be more likely to suffer from BCR.

## Background

Obesity, as a growing public health concern around the world, was linked to the development and progression of various cancers [1]. However, the association between prostate cancer and obesity, generally measured by body mass index (BMI), remained controversial [2]. Some previous studies revealed a significant association between obesity and a higher incidence of prostate cancer (PCa) [3], worse pathologic outcome [4], and higher incidence of cancer-specific mortality [5]. However, other researches failed to find such an obvious association between obesity and PCa [6, 7].

Attributed to the westernization of lifestyle and daily diet, the prevalence of obesity as well as prostate cancer was increasing rapidly in Asia (especially in China, Japan, and Korea) in the past few decades. However, the researches into the relationship between BMI and different clinicopathological characteristics and treatment outcomes in PCa patients after radical prostatectomy (RP) remained rare and inconsistent in Asia [8–10].

Therefore, we conducted a retrospective cohort study to evaluate the impact of BMI on different clinicopathological features and biochemical recurrences (BCR) after RP in Chinese PCa patients. Meanwhile, we carried out a systematic review and meta-analysis of previous studies discussing the relationship between obesity and BCR in Asia. The significance of this analysis could be far-reaching, as proper strategies towards patients with different BMI might help improve the prognosis of PCa and cost-effectiveness of postoperative treatment.

## Methods

### Study population

A total of 213 consecutive patients with clinically localized PCa underwent RP from February 2006 to December 2014 at our institute. The study was approved by the institutional review board, and written informed consent forms were signed by all patients prior to their inclusion of the study. No patient had a history of prostate surgery, hormonal therapy, chemotherapy, or radiation therapy. All patients in the study were operated on by two urologists with more than 10 years’ experience in prostatectomy. Two patients were excluded from further analysis because of missing BMI data. RP was achieved using either open (18 patients) or laparoscopic (193 patients) approach.

### Clinicopathological characteristics

Data assessed included patient age at surgery, BMI, preoperative prostate-specific antigen (PSA) level, digital rectal examination (DRE) outcomes, biopsy Gleason score, clinical stage, prostate volume (PV), prostate nodule assessed via preoperative transrectal ultrasound (TRUS), pathological Gleason score, surgical margin status, local invasion status, lymph node involvement, and postoperative follow-up PSA data. BMI (kg/m^2^) was calculated as weight (kg) divided by the square of height (m), and all patients’ height and weight information were recorded preoperatively. The patients were categorized into three groups: normal weight group (BMI <23 kg/m^2^), overweight group (BMI 23–24.9 kg/m^2^), and obese group (BMI ≥25 kg/m^2^) on the basis of Asia-Pacific criteria of obesity. Pathological evaluation of biopsy and surgical specimens was performed according to the Gleason grading system and the 2002 TNM classification. All of the biopsy and pathological specimens were obtained and evaluated in the same histopathology lab in our institute. All of the pathological results were confirmed by senior pathologists who specialized in urological pathology.

The association of different BMI categories with clinicopathological characteristics and BCR was examined. After surgery, PSA measurements were performed every 3 months for the first year and thereafter every 3 to 6 months. The date of biochemical recurrence was defined as when the serum PSA level exceeded 0.2 ng/ml, or salvage therapy was initiated even if PSA did not exceed 0.2 ng/ml.

### Statistical analysis

We assessed differences in clinicopathological characteristics across BMI categories, using the Kruskal-Wallis test for continuous variables and the chi-squared test for categorical variables. PSA and PV were analyzed after logarithmic transformations because these variables were not normally distributed. Odds ratios (OR) and 95 % confidence intervals (CI) for adverse pathological outcomes were estimated for categorical BMI using logistic regression analysis with or without adjusting for patient age, PSA, PV, clinical stage, and biopsy Gleason score. BCR-free survival was determined using Kaplan-Meier plots and log-rank test. Univariate and multivariate Cox proportional hazards regression models were adopted to find out the independent risk factor that predicted BCR. Statistical analyses were performed using the SPSS version 21 (SPSS, Chicago, IL, USA). Statistical significance was defined as a two-tailed *P* < 0.05.

### Systematic review and meta-analysis

We searched the MEDLINE and EMBASE databases on April 1, 2015, for English-written studies discussing the relationship between BCR and obesity in Asian populations. The search queries were as follows: (“obesity” or “body mass index” or “BMI”) and (prostate cancer) and (radical prostatectomy) and (“recurrence” or “progression” or “failure”).

Two investigators (Bai and Hu) assessed the eligibility of each study independently. Only clinical studies that were conducted in Asia with direct comparison between BMI and BCR following RP were incorporated in the review. We excluded reviews, editorials, meta-analysis, non-human studies, non-English-written papers, and studies for other disease settings. Data extracted from each study were as follows: title, authors, journal, publication year, study period, study population, treatment method, duration of follow-up, BMI categories, hazard ratio (HR) estimates with corresponding 95 % CI, and adjusted confounders in multivariate analysis. HR estimates across different categorical or continuous BMI were converted to the unified estimate for a 5 kg/m^2^ increase in BMI using the method stated in our previous study [11]. Unless otherwise stated, we used the most adjusted HR from each study. We assessed the heterogeneity among studies using *Q* and *I*^2^ statistics. Publication bias was examined by both Begg’s and Egger’s tests.

## Results

### BMI and clinical characteristics

The clinicopathological characteristics of patients were shown in Table 1. In total, 211 patients diagnosed with PCa with a median age of 68 years were enrolled in this study. The median BMI was 23.9 kg/m^2^. In total, 35.5 % of patients were of normal weight (BMI <23 kg/m^2^), 33.6 % of patients were overweight (BMI 23–24.9 kg/m^2^), and 30.8 % were obese (BMI >25 kg/m^2^) according to Asia-Pacific criteria. The median preoperative PSA level was 13.4 ng/ml, and the median PV was 36.0 ml. When subjects were categorized according to BMI, patients in the overweight group had more TRUS nodules compared with other two groups (chi-squared *P* = 0.049). No significant differences were observed in age, PSA, PV, and positive DRE findings.

**Table 1:** Clinicopathological characteristics of patients across different BMI categories

Variables	Total subject	BMI <23 kg/m^2^	BMI 23–24.9 kg/m^2^	BMI ≥25 kg/m^2^	*P* value^a^
No. of patients (%)	211	75(35.5)	71(33.6)	65(30.8)	
BMI (kg/m^2^)					<0.001
Median (IQR)	23.9(3.4)	21.4(2.2)	24.2(1.1)	26.4(1.9)	
Range	16.1–30.1	16.1–22.9	23.0–24.9	25.0–30.1	
Age (years)					0.373
Median (IQR)	68(8)	67(11)	70(9)	67(8)	
Range	47–86	48–86	56–83	47–81	
Preoperative PSA (ng/ml)					0.607
Median (IQR)	13.4(11.9)	13.5(15.0)	14(10.3)	11.5(13.1)	
Range	1.4–293.3	1.7–293.3	1.4–62.5	2.3–85.6	
Prostate volume (ml)					0.326
Median (IQR)	36(16)	37(13)	36(17)	35.5(18)	
Range	12–98	13–89	19–98	12–91	
Positive DRE finding (%)	68(67.6)	23(31.1)	24(33.8)	21(32.3)	0.94
TRUS nodule (%)	106(50.7)	31(41.9)	44(62.0)	31(48.4)	0.049
Clinical stage^b^ (%)					0.156
cT1	100(47.8)	41(55.4)	28(39.4)	31(48.4)	
≥cT2	109(52.2)	33(44.6)	43(60.6)	33(51.6)	
Biopsy GS (%)					0.222
≤6	82(38.9)	35(46.7)	25(35.3)	22(33.8)	
≥7	129(61.1)	40(53.3)	46(64.8)	43(66.2)	
Pathological GS (%)					0.303
≤6	65(30.8)	28(37.3)	20(28.2)	17(26.2)	
≥7	146(69.2)	47(62.7)	51(71.8)	48(73.8)	
Positive surgical margin (%)	26(12.3)	9(12.0)	4(5.6)	13(20.0)	0.039
Extra capsular invasion (%)	31(14.7)	13(17.3)	8(11.3)	10(15.4)	0.575
Seminal vesicle invasion (%)	24(11.4)	13(17.3)	4(5.6)	7(10.8)	0.083
Lymph node invasion (%)	5(2.4)	2(2.7)	1(1.4)	2(3.1)	0.797

*BMI* body mass index, *IQR* interquartile range, *PSA* prostate-specific antigen, *DRE* digital rectal examination, *TRUS* transrectal ultrasound, *GS* Gleason score

^a^All *P* values were calculated using Kruskal-Wallis test for continuous variables and chi-squared test for categorical variables

^b^The clinical stage of two patients was missing according to medical records

### BMI and pathological characteristics

Various preoperative and postoperative pathological features including biopsy Gleason score, pathological Gleason score, positive surgical margin, extra capsular invasion, seminal vesicle invasion, and lymph node involvement were assessed across BMI categories. Obese patients were at higher risk of having positive surgical margin (chi-squared *P* = 0.039). The rest of the pathological characteristics did not differ significantly across BMI categories (Table 1). In the multivariate regression model, higher BMI was an independent risk factor for a higher biopsy Gleason score (OR 1.163, 95 % CI 1.023–1.322, *P* = 0.021) and pathological Gleason score (OR 1.220, 95 % CI 1.056–1.410, *P* = 0.007) (Table 2).

**Table 2:** Logistic regression analyses of BMI categories with adverse pathological outcomes

Pathological outcomes	BMI (continuous)			BMI 23–24.9 kg/m^2^ vs. <23 kg/m^2^			BMI ≥25 kg/m^2^ vs. <23 kg/m^2^		
	OR	95 % CI	*P* value	OR	95 % CI	*P* value	OR	95 % CI	*P* value
Biopsy GS (≥7)									
Crude	1.079	0.973–1.196	0.149	1.610	0.828–3.132	0.161	1.710	0.862–3.394	0.125
Multi-adjusted analysis^a^	1.163	1.023–1.322	0.021	1.940	0.873–4.307	0.104	2.434	1.103–5.371	0.028
Pathological GS (≥7)									
Crude	1.090	0.978–1.215	0.121	1.519	0.756–3.051	0.24	1.682	0.815–3.471	0.159
Multi-adjusted analysis^a^	1.220	1.056–1.410	0.007	1.983	0.828–4.748	0.124	3.379	1.374–8.309	0.008
Positive surgical margin									
Crude	1.157	0.987–1.357	0.072	0.438	0.128–1.492	0.187	1.833	0.727–4.620	0.199
Multi-adjusted analysis^a^	1.169	0.959–1.426	0.121	0.600	0.149–2.406	0.471	1.742	0.550–5.521	0.345
Extra capsular invasion									
Crude	1.014	0.881–1.167	0.848	0.606	0.235–1.563	0.3	0.867	0.352–2.135	0.756
Multi-adjusted analysis^a^	1.145	0.950–1.381	0.156	0.945	0.273–3.267	0.929	1.687	0.551–5.161	0.360
Seminal vesicle invasion									
Crude	1.006	0.860–1.177	0.937	0.285	0.088–0.920	0.036	0.576	0.215–1.543	0.272
Multi-adjusted analysis^a^	1.070	0.865–1.323	0.532	0.414	0.104–1.641	0.209	0.840	0.251–2.810	0.778
Lymph node involvement									
Crude	1.004	0.724–1.391	0.983	0.521	0.046–5.880	0.598	1.159	0.159–8.466	0.885
Multi-adjusted analysis^a^	0.946	0.666–1.344	0.759	0.593	0.038–9.142	0.708	0.922	0.096–8.818	0.944

*OR* odds ratio, *CI* confidence interval, other abbreviations as in Table 1

^a^Adjusted for age, prostate-specific antigen, prostate volume, digital rectal examination outcomes, transrectal ultrasound outcomes, and clinical stage

### BMI and biochemical recurrence

**Fig. 1:**
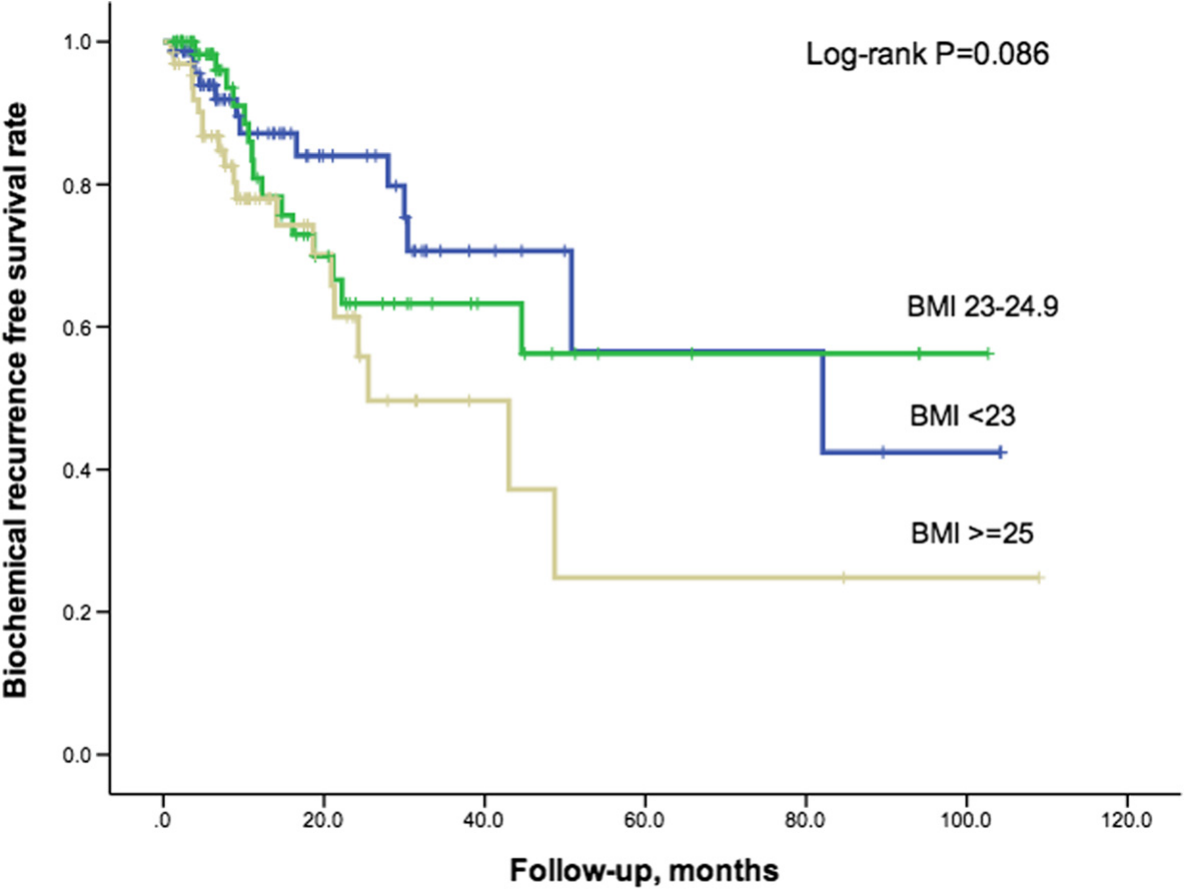
Kaplan-Meier plot for biochemical recurrence-free survival across BMI categories

Of the 211 patients, 48 (22.7 %) experienced BCR during a follow-up period of 24.5 ± 27.0 months. BCR was observed in 13 of 75 (17.3 %) patients in the normal weight group, 15 of 71 (21.1 %) patients in the overweight group, and 20 of 64 (30.8 %) patients in the obese group. The Kaplan-Meier plot indicated a weak statistically significant difference in the incidence of BCR between three groups (log-rank *P* = 0.086, Fig. 1). In univariate Cox proportional hazards analysis (Table 3), higher PSA (HR 1.509, 95 % CI 1.040–2.190, *P* = 0.03) and categorical BMI (≥25 kg/m^2^ vs. <23 kg/m^2^, HR 2.087, 95 % CI 1.033–4.215, *P* = 0.04) were both correlated with the BCR of prostate cancer. In multivariate Cox proportional hazards analysis (Table 3), higher PSA was still associated with an increasing HR trend with BCR no matter whether included BMI was a continuous or categorical variable (*P* = 0.015 and 0.011). Continuous BMI was positively associated with an increased BCR trend (HR 1.145, 95 % CI 1.029–1.273, *P* = 0.013). Categorical BMI was positively associated with an increasing HR of BCR (≥25 kg/m^2^ vs. <23 kg/m^2^, HR 2.937, 95 % CI 1.383–6.237, *P* = 0.005).

**Table 3:** Univariate and multivariate Cox proportional hazards analyses for biochemical recurrence-free survival

Variables	HR (95 % CI)	*P* value
Univariate		
Age	0.976(0.935–1.019)	0.269
BMI (continuous)	1.107(0.999–1.227)	0.053
BMI (categorical, kg/m^2^)		
<23	Reference	–
23–24.9	1.237(0.588–2.603)	0.575
≥25	2.087(1.033–4.215)	0.040
Preoperative PSA^a^	1.509(1.040–2.190)	0.030
PV^a^	1.421(0.697–2.894)	0.333
DRE	1.518(0.860–2.679)	0.15
TRUS	0.946(0.505–0.1.770)	0.862
Clinical stage	1.308(0.653–2.619)	0.448
Pathological GS (≥7)	1.133(0.597–2.152)	0.702
Positive surgical margin	0.810(0.290–2.263)	0.688
Extra capsular invasion	1.067(0.453–2.514)	0.883
Seminal vesicle invasion	1.994(0.779–5.105)	0.150
Lymph node invasion	2.835(0.872–9.218)	0.083
Multivariate model I		
BMI (continuous)	1.145(1.029–1.273)	0.013
Age	0.972(0.929–1.017)	0.225
PSA^a^	1.659(1.102–2.497)	0.015
Pathological GS (≥7)	0.991(0.512–1.917)	0.978
Positive surgical margin	0.442(0.148–1.316)	0.142
Multivariate model II		
BMI (categorical, kg/m^2^)		
<23	Reference	–
23–24.9	1.583(0.720–3.482)	0.253
≥25	2.937(1.383–6.237)	0.005
Age	0.971(0.926–1.018)	0.217
PSA^a^	1.725(1.132–2.628)	0.011
Pathological GS (≥7)	1.009(0.525–1.938)	0.979
Positive surgical margin	0.428(0.144–1.270)	0.126

*HR* hazard ratio, other abbreviations as in Tables 1 and 2

^a^Variables after logarithmic transformation

### Systematic review and meta-analysis

**Fig. 2:**
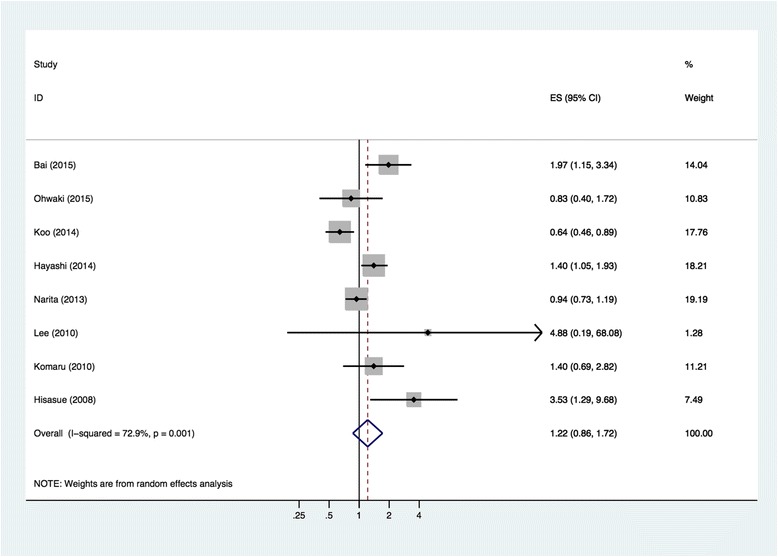
Forest plot for meta-analysis of identified eight Asian researches

Our literature search yielded 178 potentially relevant studies on the relationship between BMI and BCR of PCa after RP. After screening and full-text assessment, seven studies conducted in Asia met all eligibility criteria [8–10, 12–15] and were selected for meta-analysis along with the current study. As shown in Table 4, all eight studies followed a total of 4145 Asian PCa patients after RP from 17 to 58.2 months. Studies were conducted in Japan (*n* = 5), Korea (*n* = 2), and China (*n* = 1). The forest plot of meta-analysis showed that a 5 kg/m^2^ increase in BMI was associated with 22 % higher risk of BCR (HR 1.22, 95 % CI 0.86–1.72, Fig. 2) but failed to present statistical significance. Statistical heterogeneity was observed among the studies (*I*^2^ = 72.9 %). Begg’s (*P* = 0.536) and Egger’s (*P* = 0.215) tests did not indicate publication bias.

**Table 4:** Overview and characteristics of studies discussing BMI and biochemical recurrence of prostate cancer in Asia

Author, year [Ref]	Location	Patients	BMI (kg/m^2^)	HR (95 % CI)	HR per 5 kg/m^2^ increase of BMI (95 % CI)	Adjusted confounders^a^
Bai 2015	China	*N* = 211, 2006–2014, FUT = 24.5 months	Continuous	1.145(1.029–1.273)	1.97(1.15–3.34)	1, 2, 7, 8
Ohwaki 2015 [15]	Japan	*N* = 283, 2008–2012, FUT = 30 months	≥25 vs. <25	0.83(0.40–1.72)	0.83(0.40–1.72)	1, 2, 7, 8
Koo 2014 [10]	Korea	*N* = 880, 2005–2011, FUT = 58.2 months	>23 vs. ≤23	0.63(0.46–0.89)	0.63(0.46–0.89)	2, 5, 6, 7, 8, 14
Hayashi 2014 [12]	Japan	*N* = 703, 2002–2009, FUT = 38.4 months	Continuous	1.07(1.01–1.14)	1.40(1.05–1.93)	1, 2, 7, 8, 9, 10, 15
Narita 2013 [9]	Japan	*N* = 1257, 2001–2009, FUT = 49 months	Continuous	0.987(0.940–1.035)	0.94(0.73–1.19)	1, 2, 7, 8, 9, 10, 11
Lee 2011 [13]	Korea	*N* = 512, 2003–2009, FUT = 37.8 months	Continuous	1.373(0.720–2.326)	4.88(0.19–68.08)	1, 2, 3, 7, 8, 9, 10
Komaru 2010 [14]	Japan	*N* = 173, 1997–2007, FUT = 35 months	≥25 vs. <25	1.398(0.694–2.817)	1.40(0.69–2.82)	None
Hisasue 2008 [8]	Japan	*N* = 126, 1998–2006, FUT = 17 months	≥26.4 vs. <26.4	3.53(1.289–9.677)	3.53(1.29–9.68)	1, 2, 4, 7, 8, 12, 13

*Ref* reference, *FUT* follow-up time; other abbreviations as in Tables 1, 2, and 3

^a^Adjusted confounders: 1, age; 2, preoperative PSA; 3, prostate volume; 4, clinical stage; 5, lymphovascular invasion; 6, perineural invasion; 7, pathological Gleason score; 8, positive surgical margin; 9, extraprostatic extension; 10, seminal vesicle invasion; 11, lymph node involvement; 12, surgical period; 13, total testosterone; 14, pathological T stage; 15, adjuvant radiotherapy

## Discussion

The association between BMI and PCa is a global health concern. Unlike the US where most studies showed a positive association between greater BMI and increased aggressiveness of PCa and risk of BCR after prostatectomy [16], insufficient and contradictory data had been reported on Asian men. Two studies found that higher BMI independently contributed to biochemical failure of PCa [8, 12], while several other studies failed to confirm positive associations between BMI and adverse pathological outcomes or increased BCR [9, 10, 13–15].

As per our knowledge, the present study was the first to report results in a Chinese population, which indicated that higher BMI was an independent risk factor for a higher Gleason score, as well as an independent predictor of BCR after RP. Besides, the present study conducted a systematic review and meta-analysis of the association between BMI and BCR after RP in Asian populations. With a total of 4145 Asian PCa patients from eight studies, a positive association without statistical significance was detected between BMI and BCR.

Till now, the exact mechanisms underlying the positive association between obesity and aggressiveness of PCa were unclear. One possible mechanism for the poor prognosis of PCa among obese populations was the poor pathological outcomes of an existing tumor. Previous studies reported that obesity had a negative impact on the pathological Gleason score, positive surgical margin, extra capsular invasion, and lymph node involvement [17]. It was generally considered that BMI was positively related to an increased risk of positive surgical margins, for excessive pelvic fat might affect the surgeon’s vision and his ability to remove the entire prostate. The results of our study were partly in line with the previous researches.

Other possible mechanisms included were that higher BMI provided a favorable biological microenvironment for survival and progression of the tumor even after RP. Men with higher BMI produced a microenvironment with less testosterone and more estrogen, where PCa would be less androgen-dependent and more aggressive [18]. Besides, excessive adiposity inside the body may have contributed to the growth of tumor by secreting certain adipokines and inflammatory cytokines [19, 20]. Higher body weight was also associated with increased gene expression of diverse inflammatory transcripts in the nuclear factor kappa B pathway, promoting tumor aggressiveness [21]. Additionally, changes in the metabolic microenvironments of obese men resulted in compensatory hyperinsulinemia as well as increased levels of insulin-like growth factor 1, both of which were proved to encourage carcinogenesis and inhibit apoptosis [22]. In the present study, higher BMI was still associated with BCR even after adjusting for pathological Gleason score and positive surgical margin, which tended to support the microenvironment hypothesis.

Unlike the USA or some European countries [11], the combined HR in the present meta-analysis of Asian studies failed to adopt a positive association with statistical significance between BMI and BCR. The discrepancy between observed findings in Western and Asian populations might be explained by racial differences and differences in cancer screening and management strategy.

The average BMI in the Asian population was far lower than Western counterparts. It was suggested that only when BMI reached a certain threshold (around 30 kg/m^2^), the correlation would be significant with the increased aggressiveness of prostate cancer [13]. Most Asian obese patients failed to reach such threshold, and there might be insufficient biological changes in these men to promote aggressiveness of PCa. Besides, Asian men possessed higher body fat percentages at the same BMI compared with Western men [23]. Therefore, an Asian patient might have quite different internal biological and endocrine environment from a Western patient at a given BMI. Moreover, quite different from Western diets, Asian diets contained higher levels of phytoestrogens, which were proved to inhibit proliferative and exert pro-apoptotic effects on prostate tumor via activation of ERβ signaling [24]. Recently, more researches began to focus on the molecular basis for ethnic variation and found that several genetic alterations for prostate carcinogenesis were comparatively lower among Asian patients [25].

Selection bias might also contribute to the inconsistent results. Variations of PCa epidemiology among Asian populations could be attributed to differences in access to PSA screening, urology clinics, and available therapies [26]. PSA screening tests were not generally popularized in Asia; thus, prostate cancers in Asian patients were generally of higher stages and pathological scores before surgery. Besides, as quite a number of elder Asian patients were still concerned by surgical procedures, they would rather choose different types of androgen-deprivation therapy instead of RP as a first-line treatment towards regional prostate cancer [27]. The fluctuation of RP indications within different Asian regions might enhance heterogeneity among studies. Besides, the high heterogeneity could be further explained by different surgery methods (open or laparoscopic) and different confounders adjusted in the multivariate analyses.

The present study had some limitations. First, our study was a retrospective study in a tertiary referral hospital in China and thus may not represent the Chinese population as a whole. Second, since the patient number and follow-up time were limited, we were not able to clarify the impact of BMI on long-term prognosis. Third, the use of BMI was unable to distinguish fat from muscle. Other parameters like waist circumference, waist-to-hip ratio, and percentage of visceral adipose tissue [28] might be a better indicator for obesity.

Over the past few decades, the morbidity of obesity and PCa in Asian countries has shifted towards a more westernized high level [29]. On the contrary, Asian population-based epidemiological studies discussing the relationship between obesity and PCa prognosis were still rare. Since populations across Asian countries shared similar racial background and living customs, which were quite different from Western populations, more prospective multicenter studies would be necessary to elaborate and confirm such a relationship in Asian populations. Furthermore, other risk factors [30] such as BMI in early, middle, and late adulthood, body weight changes before or after surgery, daily diet components, exercise levels, and unrevealed biological mechanisms in Asian populations should attract wider attention from urologists and scientists.

## Conclusions

The present cohort study suggested that higher BMI was an independent risk factor for higher Gleason score, as well as an independent predictor of BCR after RP in Chinese patients. Meanwhile, the meta-analysis of Asian studies detected a positive association without statistical significance between BMI and BCR. Thus, it might be beneficial for Chinese PCa patients with higher BMI to be treated more aggressively and followed more closely after RP.

## Abbreviations

BCR: biochemical recurrence
BMI: body mass index
CI: confidence interval
DRE: digital rectal examination
HR: hazard ratio
OR: odds ratios
PCa: prostate cancer
PSA: prostate-specific antigen
PV: prostate volume
RP: radical prostatectomy
TRUS: transrectal ultrasound
